# The distribution of CTL epitopes in HIV-1 appears to be random, and similar to that of other proteomes

**DOI:** 10.1186/1471-2148-9-184

**Published:** 2009-08-04

**Authors:** Boris V Schmid, Can Keşmir, Rob J de Boer

**Affiliations:** 1 Theoretical Biology, Utrecht University, the Netherlands; 2 Academic Biomedical Centre, Utrecht University, the Netherlands

## Abstract

**Background:**

HIV-1 viruses are highly capable of mutating their proteins to escape the presentation of CTL epitopes in their current host. Upon transmission to another host, some escape mutations revert, but other remain stable in the virus sequence for at least several years. Depending on the rate of accumulation and reversion of escape mutations, HIV-1 could reach a high level of adaptation to the human population. Yusim et. al. hypothesized that the apparent clustering of CTL epitopes in the conserved regions of HIV-1 proteins could be an evolutionary signature left by large-scale adaptation of HIV-1 to its human/simian host.

**Results:**

In this paper we quantified the distribution of CTL epitopes in HIV-1 and found that that in 99% of the HIV-1 protein sequences, the epitope distribution was indistinguishable from random. Similar percentages were found for HCV, Influenza and for three eukaryote proteomes (Human, Drosophila, Yeast).

**Conclusion:**

We conclude that CTL epitopes in HIV-1 are randomly distributed, and that this distribution is similar to the distribution of CTL epitopes in proteins from other proteomes. Therefore, the visually apparent clustering of CTL epitopes in epitope maps should not be interpreted as a signature of a past large-scale adaptation of HIV-1 to the human cellular immune response.

## Background

The human immunodeficiency virus 1 (HIV-1) is a highly adaptive virus, capable of rapidly evolving its proteins to escape cellular immune responses and antiretroviral drugs (reviewed in [1] and [2]). This ability of the virus to rapidly adapt to its host has raised the question what level of adaptation to the whole human population the virus will eventually be able to reach. Currently there is no consensus on this point: on the one hand there are studies that indicate that the current HIV-1 sequences contain signatures of global adaptation [3-8], while on the other hand the virulence of the virus [9,10] as well as its predicted number of cytotoxic T cell (CTL) epitopes have remained constant over time [11].

An alternative way to study viral adaptation would be to look for tell-tale signatures of accumulated escape mutations in the virus. Yusim et. al. [12] suggested that the clustering of CTL epitopes is such a signature. They observed that regions in the virus with a low density of CTL epitopes were more variable than regions with a high epitope density. Moreover, these variable regions had a lower level of epitope precursors than the conserved regions, and contained fewer amino acids that were suitable to serve as anchor residues for MHC binding. This led to the hypothesis that HIV-1 had escaped CTL epitopes predominantly in the variable protein regions, and that large-scale adaptation of the ancestral HIV-1 sequence to the human host (or prior to that to the chimpanzee host) had resulted in the clustering of CTL epitopes that is observed in current-day HIV-1 sequences.

Another, more proximate hypothesis for the clustering of epitopes was forwarded by Lucchiari-Hartz et. al. [13]. Based on the analysis of proteasomal degradation products in HIV-1, they showed that the epitope precursors (and thus epitopes) occur preferentially in the more hydrophobic regions of HIV-1 NEF and RT proteins. They concluded that the clustering of epitopes is a generic feature of proteins, depending on the clustering of hydrophobic amino acids.

In this paper we tested whether CTL epitopes and hydrophobic amino acids in HIV-1 are significantly clustered, and compared the distribution of predicted epitopes in HIV-1 and other viruses to that of eukaryotes which are not under selection pressure to escape the cellular immune response. We discovered that for all tested protein sequences more than 95% of the epitope distributions, and more than 98% of the hydrophobic amino acid distributions were likely to be random distributions. Secondly, we discovered that there is a large amount of variation in the epitope distribution within HIV-1 proteins, similar to the amount of variation observed in eukaryote proteins of an equal length. Both findings suggest that the distribution of CTL epitopes in HIV-1 is similar to that of other proteins, and that the apparent clustering of CTL epitopes on HIV-1 epitope maps should not be interpreted as an indicator of past HIV-1 adaptation.

## Methods

### CTL epitope predictions

**Figure 1:**
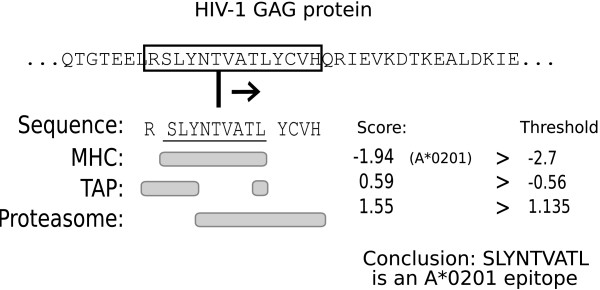
**Schematic of the MHC-pathway model**. A window of 14 amino acids is slided across a protein. Each of these '14 mers' consist of a N-terminal flanking region of 1 amino acid, a 9 mer epitope candidate and a C-terminal flanking region of 4 amino acids. Beneath the 14 mer, the parts of the peptide are marked which are used by the MHC, TAP or proteasome predictors. Applying the 14 mer to the MHC, TAP and proteasome predictors results in three different scores. Only if each of these scores is higher than a fixed threshold, the epitope candidate is predicted to be a CTL epitope for the MHC allele under consideration.

There are several algorithms [14-17] available that can predict the location and binding specificity of CTL epitopes in protein sequences. In this study we use the MHC-pathway model [14], which allows us to screen all possible peptide fragments of 14 amino acids within a particular protein for their ability to be correctly processed by the proteasome and transporter associated with antigen processing (TAP), and presented by the MHC class I molecules. Peptide fragments that can be correctly processed by all three steps are subsequently marked as CTL epitopes (Fig. 1). An extensive analysis of the quality of these predictors can be found in the methods section of Schmid et. al. [11]. A brief synopsis is that 81–97% of the predicted CTL epitopes are indeed CTL epitopes [18,19].

The threshold values for the proteasome and TAP predictors (Fig. 1) were derived by applying the MHC-pathway model to a large bacterial protein data set and selecting the threshold values which corresponded to the estimated specificity of the proteasome (33%) and TAP (76%) [20]. For the MHC-binding predictions we used the default threshold of -2.7, which corresponds to an IC50 threshold of 500 nM [21,22]. The predictors used in this paper are available through a web interface (http://www.iedb.org 2006-01-01 version), and consist of an immunoproteasome cleavage predictor, a TAP transport predictor and 34 different MHC class I alleles binding predictors (18 for human leukocyte antigen (HLA)-A alleles and 14 for HLA-B alleles). Based on our previous work with these predictors [11], we excluded the A*3002 and the B*0801 MHC class I binding predictors for being too unspecific or specific, respectively.

### Describing epitope clusters

**Figure 2:**
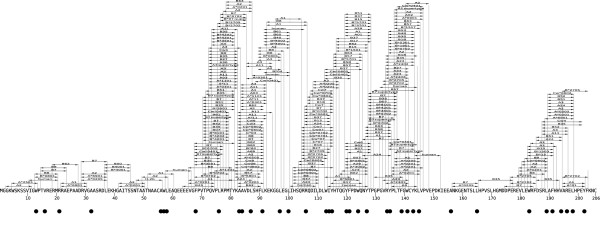
**Example of an epitope map of HIV-1 NEF and its transformed version (black dots)**. The epitope map was retrieved from http://www.hiv.lanl.gov (Jul 31 2008 version). This specific epitope sequence (an unspecified HXB2 variant) carries 52 predicted epitope precursors, of which 38 are predicted to be epitopes (binding at least one of the 32 MHC class I allele binding predictors).

CTL epitopes are traditionally defined by their amino acid sequence, the start and end-point of the sequence mapped onto a reference sequence, and the MHC class I allele that they bind to. Based on this definition, a natural way to visually present CTL epitopes is an epitope map (Fig. 2). However, for the statistical study of the clustering of CTL epitopes it is more practical to reduce the position of a particular epitope to a single point. In this paper we have opted to use the C-terminal amino acid of CTL epitopes to position an epitope in a protein sequence, as the C-terminal amino acid is the most defining property of an epitope: amino acid substitutions at the C-terminal have a large effect on proteasomal cleavage, TAP transportation and MHC binding [23,24] (Fig. 1). One effect of this transformation is that epitopes are only defined by their position in a protein, and no longer by the MHC allele(s) that they bind. Thus, epitopes that only differ in the MHC that they bind to will be reported as a single epitope. Although this transformation makes it possible to perform a clustering analysis on the spatial distribution of CTL epitopes, it might destroy an evolutionary signature that is contained in the number of MHC alleles that bind to individual epitope precursors. We discuss this in the final section of the paper.

### Clustering methods

Methods to describe the degree of clustering of sequential events or spatial locations have been developed in a large number of scientific fields, ranging from astronomy to ecology and economics. These methods consider two features of a clustering: the 'intensity' and the 'grain'. The intensity reflects the difference in object density between the rich and poor regions, and the grain describes how frequently rich and poor regions alternate [25]. In this paper we will use two methods: the cumulative binomial probability (CBP) method [26], and the Hopkins and Skellam index (H&S) [25,27].

**Figure 3:**
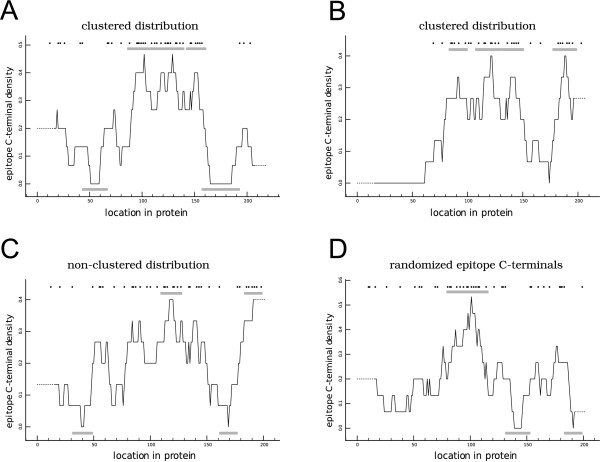
**Example of the cumulative binomial probability profiles of 3 HIV-1 NEF protein sequences**. Each profile features the position of predicted epitope C-terminals in the protein sequence (black dots), a running average of the C-terminal density (black line, window size 15), and the epitope-rich and/or epitope-poor regions (grey blocks) (see Eq. 1). (A + B) The distribution of CTL epitopes in the protein sequence in panel A (accession number: DQ351225) has a low probability to arise from a random distribution of CTL epitopes, based on the cumulative binomial probability method. The protein sequence in panel B (accession number: AJ233029) has a low probability to arise from a random distribution of CTL epitopes, based on the Hopkins and Skellam index of clustering, but less so according to the CBP method. (Panel A, CBP: *p*_*rich *_= 0.0056, *p*_*poor *_= 0.0062; H&S: p = 0.015. Panel B, CBP: *p*_*rich *_= 0.06, *p*_*poor *_not computable for a window size of 15 or smaller (see Methods); H&S: p < 0.001.). (C) The distribution of CTL epitopes in the protein sequence in panel C (accession number:AY905390) has a high probability to arise from a random distribution. CBP: *p*_*rich *_= 0.7026, *p*_*poor *_= 0.5328; H&S: p = 0.195. (D) The same sequence as in Panel C, but with the positions of the epitope C-terminals randomized.

Regarding the cumulative binomial probability method (CBP) [26]: this method can be used to determine whether a particular amino acid lies in a region that is rich, poor or neutral in epitope density (Eq. 1). For example, to determine whether a particular amino acid is located in an epitope-rich region, one first counts the number of epitope C-terminals (*e*) in a window of size *w*, and then, based on the average epitope-density in the protein (*f*), one calculates the chance of finding *e *or more epitope C-terminals in a window of that size (*i *= *e, i *= *e *+ 1, *i *= ...,*i *= *w*). If this chance *P *falls below a certain threshold (0.05 in this paper), all amino acids in that window are marked as belonging to an epitope-rich region. The same approach can be used to determine which amino acids belong to epitope-poor regions. The CBP method makes it possible to objectively determine the location of epitope-rich and epitope-poor regions in a protein. These locations can be plotted to generate a CBP profile (see Fig. 3).(1)

Regarding the Hopkins and Skellam index (H&S) [25,27]: this method is based on the observation that in a fully random distribution (of an infinite size), the distance from a starting point to the nearest object of interest is not influenced by the presence or absence of such an object at the starting point. In an over-dispersed distribution, the presence of an object at the starting point will mean that the nearest object is on average further away than when starting at a random location, while in a clustered distribution, the reverse is true. The ratio is calculated as the sum of squared distances from a random point to the nearest object (*d*_*r*_) to the sum of squared distances from a random object to the nearest object (*d*_*o*_). When the number of *d*_*r *_and *d*_*o *_measurements are not equal, the sum of squared distance of *d*_*r *_and *d*_*o *_should be divided by the number of *d*_*r *_measurements (*n*) and *d*_*o *_measurements (*m*), respectively. The ratio will be a number (*R*) between 0 for perfectly over-dispersed distributions, and infinity for fully clustered distributions (Eq. 2). In this way the distribution of epitopes within a protein can be characterized by a single ratio. In this paper we normalized the range of the H&S index in such a way that the index runs from 0 to 2, rather than from 0 to infinity, by translating any score above one to 2 - (1/*score*).

Both the CBP method and the H&S index take into account the 'intensity' and the 'grain' of epitope distributions and correct for the epitope density of the protein. One difference between the two methods is that the latter gives a higher clustering score to coarse grained distributions, whereas the former favors fine-grained distributions (see results, section 'No clustering of epitopes').(2)

### Statistical testing

The significance of both clustering measures can be tested with permutation tests [28-30]. Permutations are created by randomizing the positions of the epitope C-terminals in the protein that is under scrutiny. The p-value of the test is the fraction of cases in which the randomized sequence has an equal or more extreme outcome than the original sequence. In the case of the CBP method the outcome was measured as the fraction of the protein that is part of an epitope-rich region (or epitope-poor region, when studying those). In the case of the H&S index, the outcome was measured as the absolute difference of the index score from 1.0 (the expected score for a random distribution).

### Hydrophobicity

In order to determine whether hydrophobicity is clustered, we calculated the clustering of the top 4 hydrophobic amino acids (Leu, Ile, Phe, and Trp, according to both the HPLC pH 7.4 scale [31] used by Lucchiari-Hartz et. al. [13], and the consensus scale [32]), with the CBP method and the H&S index. This is somewhat different from the more common approach of calculating the running average hydrophobicity and setting one or two thresholds to determine the hydrophobic and hydrophilic areas of a protein, (as was done in the study of Lucchiari-Hartz et. al. [13]). However, it has the advantage that we can use the same method for determining epitope clustering and hydrophobic amino acid clustering.

### Data sets

The public data sets used in this paper originate from a variety of sources. Pre-aligned HIV-1 and HCV data (size: 13093 and 8886 proteins, respectively) were downloaded from the Los Alamos laboratories (http://www.hiv.lanl.gov, http://www.hcv.lanl.gov), and the influenza data set (size: 47194 proteins) was downloaded from Biohealthbase (http://www.biohealthbase.org, under Influenza Virus, Database Search, Sequence), by selecting for all available proteins from human influenza type A, B or C. A Human [33] (IPI.human.prot, size: 72082 unique proteins), Drosophila (size: 23694 unique proteins), and a Yeast proteome (size: 5863 unique proteins) were downloaded from Integrate http://www.ebi.ac.uk/integr8/. All data sets were downloaded on 13 Aug 2008.

The public HIV-1 and HCV data sets are already curated, and do not contain multiple clones from one isolate, or multiple sequences from a single person. Furthermore, very similar groups of sequences (based on phylogenetic tree analysis) are also reduced to a single sequence. In all three eukaryote proteomes, only unique protein sequences are used.

The ancestral HIV-1 clade B sequence [34] can be downloaded at http://www.hiv.lanl.gov.

## Results and discussion

### Imprints of immune evasion in HIV-1

**Figure 4:**
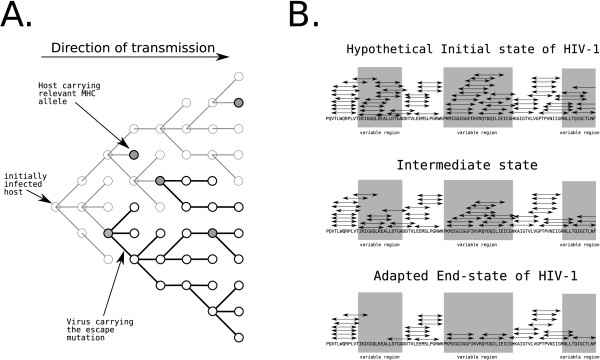
**(A) Schematic of a transmission network of HIV-1 through the human population**. An escape variant of a particular CTL epitope can rapidly become the consensus sequence if reversion of the escape happens little or not at all. Of the 50 hosts (circles), only 5 hosts (filled circles) carry an MHC allele that can bind the epitope. The black circles represent hosts that are infected by the escape variant of the virus, but lack the relevant MHC allele. Grey lines represent transmission of the wildtype virus, whereas black lines represent transmission of the escape variant. (B) Schematic of the accumulation of escapes in the variable protein regions. When escape mutations occur more often, or reversion happen more slowly in variable protein regions (gray shaded areas), and the number of accumulated escape mutations is large enough, a clustering of CTL epitopes (plotted as arrow-delimited lines) is to be expected. One underlying assumption is that the variable and conserved protein regions are larger than a few amino acids in size [12].

HIV-1 is capable of maintaining escape mutations to CTL epitopes in the absence of immune selection pressure of a MHC-matched host [35,36], and thus escape variants of HIV-1 can become the consensus HIV-1 sequence [3,4]. Escape variants with a low fitness cost or compensatory mutations revert slowly or not at all [8,37], and could quickly accumulate in the virus [4,36]. Fig. 4A sketches the fast spread of a non-reverting escape variant in a hypothetical transmission network. Even though the hosts which carry an MHC allele that can bind to the CTL epitope are not optimally positioned in the transmission network, it only takes a few transmissions before the majority (54%) of the hosts carries the escape variant of the virus.

Yusim et. al. [12] studied the apparent clustering of CTL epitopes in HIV-1 epitope maps, and found a negative correlation between CTL epitope density and sequence variability in HIV-1. Based on the paucity of epitope precursors and suitable MHC anchor residues in the variable protein regions, Yusim et. al. [12] concluded that the lack of epitopes in the variable regions was a signature of immune evasion of the virus. The conserved protein regions were assumed have more constraints related to protein function, and the virus would have fewer viable options to generate escape variants in these regions [12], because the escapes made in these conserved regions would carry a higher fitness cost [38,39]. As a result, Yusim et. al. [12] argued that the accumulation of escape mutations would be slower, and reversion of escape mutations faster in conserved protein regions than in variable regions. These ideas are depicted in Fig. 4B. This difference in the rate of accumulation of escape mutations between the variable and conserved protein regions is expected to result in a clustering of CTL epitopes once the virus has accumulated a substantial number of escape mutations. Taken together, Yusim et al. [12] concluded that the apparent clustering of CTL epitopes in epitope maps was a signature of a large-scale adaptation of HIV-1 to the human population.

### Clustering in epitope maps

The first reports that the CTL epitopes of HIV-1 occurred in clusters [39-41] were published only a few years after the discovery of HIV-1 CTL epitopes themselves [42,43]. However, the degree of clustering of CTL epitopes has never been tested rigorously, perhaps because the method by which epitope positions are visualized in epitope maps strongly suggests that a clustering exists (Fig. 2). Here we list a number of reasons why epitope maps may give an unjust impression of clustering:

1. The epitope map is a compilation of the CTL epitopes found in a large number of sequences. Amino acid variants of the same epitope are all depicted at the same position of the reference sequence, but never occur simultaneously in a single HIV-1 sequence.

2. CTL epitopes that have not been mapped precisely to their minimal length can end up occurring more than once on the epitope maps as N- or C-terminal extended versions of an epitope.

3. Epitope precursors are expected to be generated at roughly 25% of the positions in a protein [20]. The large polymorphism in MHC class I alleles makes it likely that a single epitope precursor binds to multiple MHC alleles [44]. Therefore, the absence or presence of an epitope precursor at a certain position results in either zero or many epitopes reported at that position.

4. CTL epitopes on the maps are vertically ordered to be non-overlapping. This representation results in empty corridors between large slanted towers of epitopes. The corridors need not correspond to epitope-poor regions, but are a visual effect of the representation.

All four reasons listed amplify the difference between epitope-rich and epitope-poor regions, with the result that a strong clustering of CTL epitopes appears to exist. Most of these reasons have already been listed by the scientists maintaining the epitope maps at http://www.hiv.lanl.gov, but the suggestive effect on epitope clustering is not mentioned explicitly. We removed these amplifications by using CTL epitope predictors (removing 2.) on a per-sequence basis (removing 1.), and by only plotting their C-terminal position (remove 3. and 4.). This transformation results in a binary pattern of epitope C-terminal positions: each position being either an epitope or a non-epitope (see Fig. 2, Methods). Although necessary for a meaningful analysis of the clustering of CTL epitopes, the transformation could potentially destroy an evolutionary signal if HIV-1 has evolved to have fewer MHC alleles binding per epitope precursors in certain protein regions. We will consider this option in the discussion.

We use epitope predictors rather than lists of known CTL epitopes, as the predicted epitopes are less influenced by an 'attention bias' than experimentally defined CTL epitopes. An attention bias can be caused by researchers focusing on hot topics or building on previous work. Assarsson et. al. [45] showed that as a result of this bias, certain protein regions in Influenza are mistakenly classified as CTL epitope-rich or poor.

Epitope predictors, such as the MHC-pathway algorithm, predict proteasomal cleavage, transporter associated with antigen processing (TAP) and MHC class I binding [14] for all peptide fragments within a protein. Those fragments that can be processed by each of these steps are predicted to be CTL epitopes (see Fig. 1). As the MHC-pathway algorithm has been tested extensively [21] and has proven to have a high reliability [18,19] (see Methods), it allowed us to avoid a possible 'attention bias' in HIV-1 protein regions or strains.

### No clustering of epitopes

We applied two distinct methods of measuring distributions to the epitope distribution of HIV-1 proteins. The first method divides proteins into epitope-rich, epitope-poor, and neutral regions, based on the cumulative binomial probability (CBP) [26] of having *e *or more amino acids predicted as an epitope C-terminal in a window of size *w *(Eq. 1). The second method is the Hopkins and Skellam (H&S) index [25,27], which compares the average distance from an epitope to its nearest epitope with the average distance from a random amino acid to the nearest epitope within proteins (Eq. 2). Both methods are subjected to permutation tests in order to establish per protein the significance of its distribution of CTL epitopes. A more extensive discussion of these methods and the permutation testing is available in the Methods section.

Using both the CBP method and the H&S index, we find protein sequences in HIV-1 with CTL epitope distributions that are likely to be random, as well as distributions that are likely to be clustered. We visualized a few of these protein sequences using CBP profiles (Fig. 3), as well as a sequence in which the positions of the CTL epitopes were randomized. Note that each of the four visualized sequences, including the randomized one, contain epitope-rich and/or epitope-poor regions. Thus, the mere presence of epitope-rich or epitope-poor regions in a protein does not need to imply that adaptation has occurred in that region.

We analyzed the predicted CTL epitope distribution in a data set of 11017 HIV-1 proteins from the Los Alamos HIV-1 Sequence compendium with the CBP method, and found that in 99% of these sequences the fraction of epitope-rich regions was not significantly different from random (p < 0.001, permutation test). Only 158 sequences had a larger fraction of epitope-rich regions than likely to arise in a random distribution of CTL epitopes. These 158 sequences predominantly occurred in two specific HIV-1 proteins: HIV-1 VPU (79×) and HIV-1 ENV (74×). Changing the window size *w *from 15 to 9 or 23 shifted the number of significant sequences towards VPU or ENV, respectively, but did not affect the overall lack of significantly clustered CTL epitopes. Similar to what we found for the epitope-rich regions, only 153 sequences in HIV-1 had a larger fraction of epitope-poor regions than expected, most of which occurred in VPU (135×).

**Figure 5:**
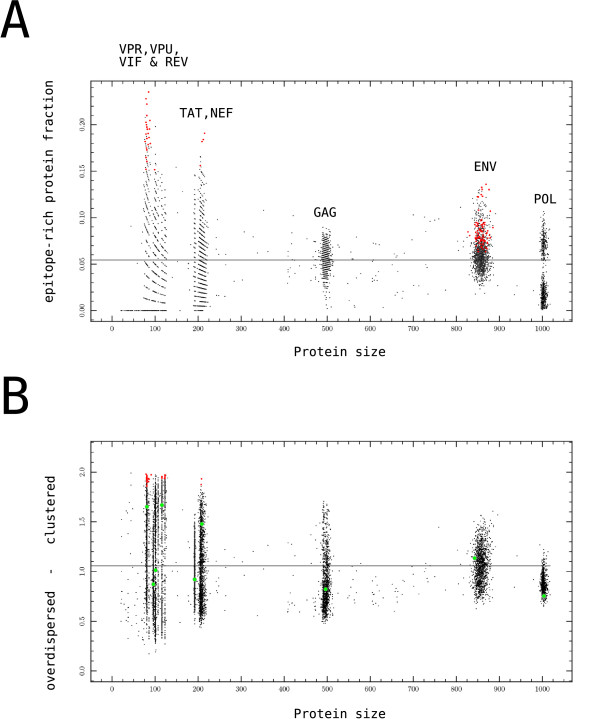
**Analysis of HIV proteomes**. Each protein sequence is plotted on the horizontal axis according to its size (dots). Red dots are significant (p < 0.001). Above each large cloud of sequences, the corresponding protein name is denoted. Sequences in between clouds are likely to be truncated version of larger proteins that were not pruned from the data set. The grey line denotes the average score. (A) The CBP method yielded only 153 out of 11017 sequences whose epitope distribution was unlikely to be random (less than 2%). (B) The H&S index yielded 68 sequences unlikely to be random (less than 1%). Marked with green dots are the predicted ancestral sequence of HIV-1 clade B proteins.

The H&S index gives a similar result as the CBP method: only 68 HIV-1 protein sequences (0.6%) had a predicted epitope distribution that is significantly more clustered than expected from a random distribution, and most of these occurred in the VPU protein (55×). The distribution of CTL epitopes in the predicted ancestral HIV-1 clade B sequences [34] (green dots, Fig. 5B) is also not significantly different from random. The fact that we found HIV-1 ENV and VPU to be the proteins in which some sign of clustering occurred would not be what one expects if the clustering would be due to adaptation. The GAG protein would have been a more obvious candidate, both for its early presentation on the cell surface [46] and its immunodominant CTL epitopes [47].

There is a remarkable large variation between sequences of the same protein in both the CBP and the H&S methods. Sequences range from being devoid of epitope-rich protein regions to having 20% of their amino acids belonging to an epitope-rich region in the CBP method (Fig. 5A). The same holds for epitope-poor regions (data not shown). Sequences range from a highly clustered to a moderately over-dispersed H&S index score (Fig. 5B), even when reducing the data set to specific HIV-1 clades (data not shown). Apparently, even within closely related sequences, relatively small amino acid differences can cause large variations in the degree of CTL epitope clustering, up to a degree that the H&S index score variation for most of HIV-1's proteins is similar to that of randomized proteins of the same size. The POL and TAT proteins displayed less variation than expected for their protein size.

When comparing the significantly clustered sequences predicted by both methods, we find an overlap of 21%. While this is significantly higher than the expected overlap of 1.4% (Fisher's exact test, p = 0.0008), the two methods are often in disagreement whether a sequence is significantly clustered. This could be due to the difference in how both methods valuate the 'grain' of a pattern (i.e. how frequently rich and poor regions alternate). The H&S index valuates coarse-grained clusters (Fig. 5) above fine grained clusters, whereas the CBP method does the opposite.

### Comparison between species

Although CTL epitopes in HIV-1 are typically randomly distributed (Fig. 5), a direct comparison of the CTL epitope distributions between virus and eukaryote proteomes might reveal a difference between both groups that is due to immune selection pressure. We included two additional virus sequence data sets in the analysis, namely the Hepatitis C Virus (HCV) and Influenza, and picked three eukaryote proteomes: the human *Homo sapiens*, fruitfly *Drosophila melanogaster *[48] and yeast *Saccharomyces cerevisiae *proteome. The latter two are proteomes that normally do not come into contact with the human antigen presentation pathway, and should therefore not be adapted to it.

**Figure 6:**
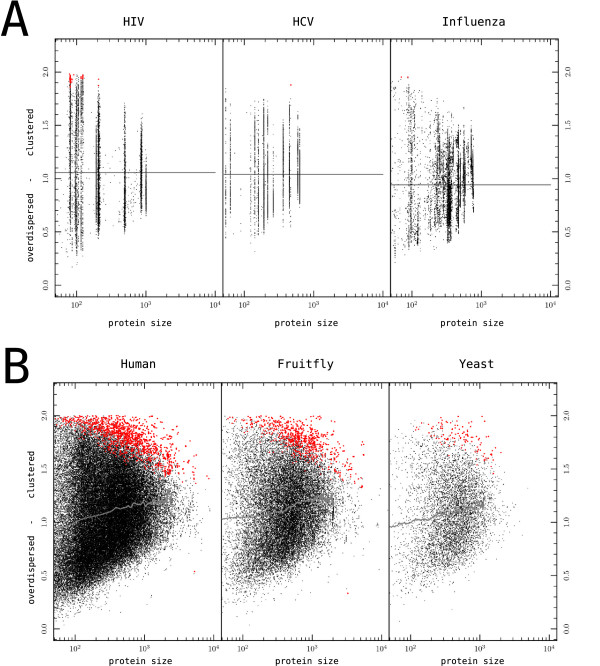
**The H&S index for six proteomes, plotted against protein length**. The top three panels display virus proteome data sets, and contain multiple sequences per protein. Each vertical cloud corresponds to another protein. The bottom three panels display the proteomes of Human, Drosophila and Yeast. (A) Grey line: average index score taken over all sequences. (B) Grey line: running average of the index score (window size of 70). (A + B) Red dots: protein sequences whose epitope distribution is significantly unlikely to be random (p < 0.001, permutation test).

The distribution of predicted CTL epitopes in HCV and Influenza was similar to that of HIV-1. The vast majority of sequences featured a random distribution of CTL epitopes (> 99%), and a large amount of variation in H&S clustering score per protein, just as seen in HIV-1 (Fig. 6A). Although in all three viruses some proteins tended towards clustering, and others towards over-dispersion of epitopes, we have not been able to detect a pattern in these tendencies. One difference between the viruses was that the small fraction of significantly clustered sequences was somewhat higher in HIV-1 (0.6%) than in HCV (0.01%) and Influenza (0.00%), but as we are comparing many related copies of only a small number of proteins, this difference could well be due to chance.

A comparison of the three eukaryote proteomes revealed that their CTL epitope distributions are remarkably similar to each other. In all three proteomes there is a steady trend towards clustered epitope distributions with increasing protein size (Fig. 6B, grey line). It could be that these significant proteins contain more structural motifs and repeating elements that the other proteins, and that these motifs influence the epitope distribution [49,50]. The percentage of significantly clustered sequences (H&S) is a few percentage-points higher than in the viruses (Human: 1.9%, Drosophila: 3.4%, Yeast: 1.9%, at a p < 0.001, permutation test), but is still only a small percentage of all sequences.

**Figure 7:**
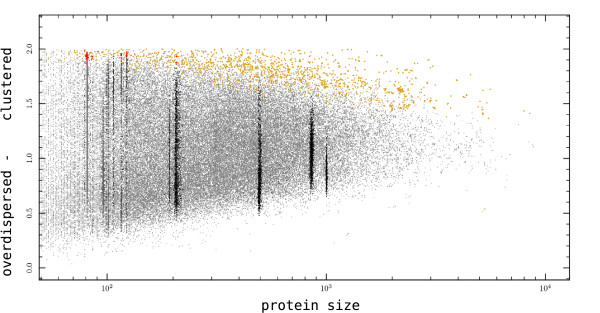
**An overlay of HIV-1 proteins on top of the human proteome**. The degree of clustering of CTL epitopes in proteins is determined by the H&S index and plotted against protein length. Significantly clustered sequences are denoted in yellow dots for the human proteome, and in red dots for HIV-1 (p < 0.001, permutation test). The index scores of HIV-1 fall within the range found in human proteomes, suggesting that the epitope distribution pattern of HIV-1 is not extraordinary.

An overlay of HIV-1 on the human proteome shows that the H&S clustering scores for HIV-1 proteins fall within the range of scores for human proteins (Fig. 7). The variation within HIV-1 proteins spans about the same range as proteins of comparable size in the human proteome. This is surprising, as the sequences within HIV-1 proteins are closely related to each other, and would therefore be expected to have a smaller range of clustering scores (for the POL and TAT protein this seems to be partially true).

Summarizing, the CTL epitopes of > 99% of HIV-1, HCV and Influenza sequences were found to be randomly distributed. A comparison between viral and eukaryote proteomes showed no qualitative differences in the epitope distribution between the two groups that would point towards the adaptation of viruses to the human host.

### No clustering of hydrophobic amino acids

An alternative hypothesis on epitope clustering that was forwarded by Lucchiari-Hartz et. al. [13], challenged the idea that the distribution reflected the adaptation of HIV-1 to its new host, and suggested that the clustering of CTL epitopes merely mirrored the clustering of hydrophobic amino acids. As the proteasome, the TAP, and many of the MHC alleles favor hydrophobic amino acids at or near their C-terminal end [20,51,52], a clustering of hydrophobic amino acids would result in a clustering of epitope precursors, and subsequently results in a clustering of CTL epitopes.

Our results thus far dispute the idea that CTL epitopes are clustered, as we found the epitope distribution in the vast majority (> 99%) of protein sequences to be not different from a random distribution. Therefore we wondered if hydrophobic amino acids are truly clustered in proteins, and repeated our clustering analysis for hydrophobic amino acids. By taking the four most hydrophobic amino acids (Leu, Ile, Phe and Trp [31,32]), we could construct binary maps similar to the transformed epitope maps of Fig. 2.

**Figure 8:**
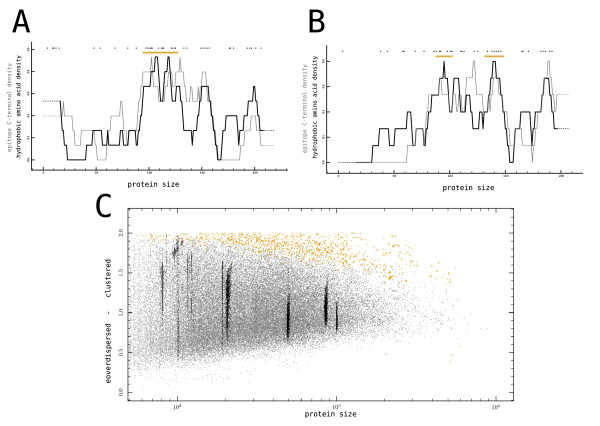
**(A + B) CBP profiles of the hydrophobic amino acids (Leu, Ile, Phe, and Trp) of the same two HIV-1 NEF sequences as profiled in Fig. 3A and Fig. 3B**. Hydrophobic amino acids (black dots) and hydrophobic areas (orange blocks) are depicted above the running average (window size 15) of hydrophobic amino acid density (black line) and of the epitope C-terminal density (grey line). (A) Both the fraction of the sequence that is part of a hydrophobic region (14%), and the H&S index score (1.48) are likely to occur at random (CBP: *p*_*rich *_= 0.099, H&S: *p *= 0.095). (B) Both the fraction of the sequence that is part of a hydrophobic region (15%), and the H&S index score (0.758) are likely to occur at random (CBP: *p*_*rich *_= 0.047, H&S: *p *= 0.576). (C) An overlay of HIV-1 proteins on top of the human proteome. The degree of clustering of hydrophobic amino acids is determined by the H&S index and plotted against protein length. Significant sequences (i.e. *p *< 0.001, permutation test) are plotted as red dots for HIV-1 (only 5 out of 11039), and yellow dots for Human (1195 out of 70269).

We found that nearly 100% of the protein sequences in HIV-1 had no significant clustering of hydrophobic amino acids in their primary structure (Fig. 8C). The biophysical community is somewhat divided on this point: depending on the method used and the subset of proteins studied, both random [53] and non-random distributions [49,54] have been reported. We agree that some signs of non-randomness is to be expected in the distribution of amino acids in proteins, as common protein structures like *a *helices, and *β *sheets have a certain periodicity in their use of hydrophobic amino acids [49]. However, because we find so few proteins in which hydrophobic amino acids are significantly clustered, it seems safe to conclude that the effect of protein structure on the distribution of hydrophobic amino acids is rather subtle.

As was shown previously by Lucchiari-Hartz et. al. [13], hydrophobic amino acids and the location of epitope C-terminals in HIV-1 correlate. This is visible in CBP profiles (Fig. 8A, 8B, black and grey lines), and statistically confirmed in the overlap between sequences with significantly clustered epitope distributions and significantly clustered hydrophobic amino acid distributions in the human proteome (Fisher's exact test, p < 0.0001, n = 69685). Summarizing, we find that epitope-poor regions correlate with hydrophilic regions, but that neither epitopes nor hydrophobic amino acids are distributed in a way that is significantly different from a random distribution.

## Conclusion

We showed that the vast majority (>99%) of HIV-1, HCV and Influenza proteins has a predicted CTL epitope distribution that is indistinguishable from a random distribution (Fig. 5). Additionally, the distribution of hydrophobic amino acids in these proteins is also likely to be random (Fig. 8C). These findings cast doubt on two recent hypothesis in which it was argued that the clustering of CTL epitopes in HIV-1 proteins is the product of virus adaptation [12], or the result of clustered hydrophobic amino acids [13], respectively.

To further investigate if there was any sign of evolution in the distribution of CTL epitopes in viruses, we compared three virus proteomes to the proteomes of Human, Drosophila and Yeast (Fig. 6). We found that the epitope distribution in HIV-1 proteins, as measured by the Hopkins & Skellam index score, is not extraordinary and falls within the range of proteins of comparable size in the human proteome (Fig. 7). Remarkably, the variation in epitope distribution that exists for any HIV-1 protein when sampling the virus from many hosts, is as broad as the whole range of distributions found between all eukaryotic proteins of a comparable size as the sampled HIV-1 protein. Such a large amount of variation in epitope distributions is not what one would expect if HIV-1 has been undergoing large-scale adaptation to the human population. If HIV-1 had been globally accumulating the same CTL epitope escapes in its variable protein regions [12], the distribution of CTL epitopes within HIV-1 viruses should be converging towards one particular distribution.

**Figure 9:**
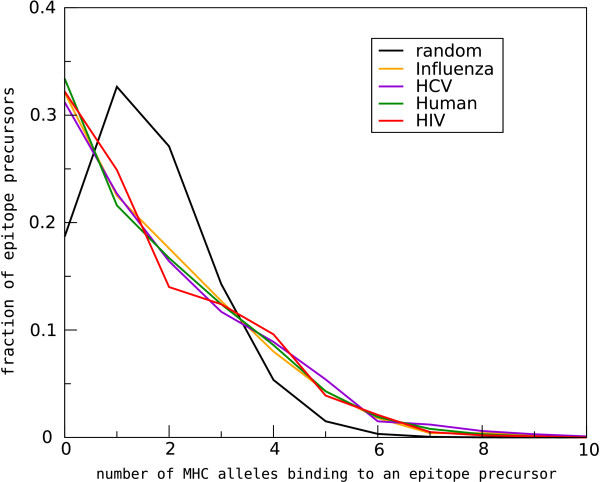
**MHC alleles are clustered over epitope precursors**. A single epitope precursor will be predicted to bind somewhere between 0 and 32 HLA molecules (as for 32 MHC alleles there are SMM binding predictors available). In a situation where MHC alleles are randomly distributed over epitope precursors, each MHC allele has a chance to bind to an epitope precursor with the same chance as the specificity of that MHC allele (which for a threshold of IC50 500 nM ranged from 0.5% to 13% of the epitope precursors). Given a random distribution, 18% of the epitope precursors are expected to bind to none of the available MHC alleles, 33% to bind a single MHC allele, and 49% to bind 2 or more MHC alleles (black line). In contrast with the random distribution, the predicted distribution for HIV-1 shows a higher percentage of epitope precursors that bind no epitope precursors, fewer than expected epitope precursors that bind between 1 and 3 MHC alleles, and more than expected epitope precursors that bind to 4 or more MHC alleles (red line). Not only HIV-1, but also HCV, Influenza, and the Human proteome follow this pattern.

The transformation that we applied to analyse the spatial distribution of CTL epitopes in proteins (discussed in section 4.2 and 5.2) could have destroyed one possible fingerprint of HIV-1 adaptation, namely that in its variable regions, HIV-1 has adapted to select for epitope precursor to which only a limited number of MHC alleles can bind [12]. We found that, compared to a random distribution, a larger fraction of epitope precursors is predicted not to bind to any of the 32 studied MHC alleles (33%) than expected. Furthermore, the number of epitope precursors that bind to 1, 2 or 3 MHC molecules is underrepresented, whereas the number of epitope precursors that bind to 4 or more MHC molecules is over-represented in HIV-1. This pattern of under- and over-representation strongly suggests that the number of MHC alleles that can bind to a particular amino acid sequence is clustered. However, this pattern is not only observed for HIV-1 proteins, but also for HCV, Influenza, and the Human proteome (Fig. 9), which suggests that the clustering of MHC alleles over epitope precursors reflects patterns in the binding preferences of MHC alleles, and not as much a fingerprint of HIV-1 adaptation to its human host.

Whether or not HIV-1 is currently adapting to the human population is debated in the literature, and investigated with the help of a variety of methods [3-10,12]. We have previously reported that HIV-1 did not show any large-scale adaptation to the cellular immune response over the last three decades [11], using HIV-1 population sequence data sets and CTL epitope predictors. In this paper we show that the distribution of predicted CTL epitopes in HIV-1 appears to be random, and is similar to the distribution of CTL epitopes in organisms that are not under selection pressure to escape the human antigen presentation pathway. Therefore we conclude that the visually apparent clustering of CTL epitopes in epitope maps should not be interpreted as a signature of a past large-scale adaptation of HIV-1 to the human cellular immune response.

## Authors' contributions

BVS, CK and RJB all contributed to conception and design of the study. BVS analysed the data, and performed the statistical testing. BVS, CK and RJB all contributed to the interpretation of the results. BVS drafted the manuscript and created the figures. CK and RJB extensively commented on the drafts and figures. All authors read and approved the final manuscript.
